# Modeling the Mental Health Practice Change Preferences of Educators: A Discrete-Choice Conjoint Experiment

**DOI:** 10.1007/s12310-013-9110-8

**Published:** 2013-10-30

**Authors:** Charles E. Cunningham, Melanie Barwick, Kathy Short, Yvonne Chen, Heather Rimas, Jenna Ratcliffe, Stephanie Mielko

**Affiliations:** 1 Hamilton Health Sciences, McMaster Children’s Hospital, McMaster University, Hamilton, ON L9C 7N4 Canada; 2 Hospital for Sick Children, University of Toronto, Toronto, ON Canada; 3 Hamilton-Wentworth District School Board, Hamilton, ON Canada; 4 McMaster University, Hamilton, ON Canada; 5 Department of Psychiatry and Behavioural Neurosciences, Faculty of Health Sciences, The Jack Laidlaw Chair in Patient-Centred Health Care, McMaster University, Hamilton, ON Canada

**Keywords:** Evidence-based practice, Mental health, Schools, Implementation, Conjoint analysis

## Abstract

Schools are sometimes slow to adopt evidence-based strategies for improving the mental health outcomes of students. This study used a discrete-choice conjoint experiment to model factors influencing the decision of educators to adopt strategies for improving children’s mental health outcomes. A sample of 1,010 educators made choices between hypothetical mental health practice change strategies composed by systematically varying the four levels of 16 practice change attributes. Latent class analysis yielded two segments with different practice change preferences. Both segments preferred small-group workshops, conducted by engaging experts, teaching skills applicable to all students. Participants expressed little interest in Internet options. The support of colleagues, administrators, and unions exerted a strong influence on the practice change choices of both segments. The *Change Ready* segment, 77.1 % of the sample, was more intent on adopting new strategies to improve the mental health of students. They preferred that schools, rather than the provincial ministry of education, make practice change decisions, coaching was provided to all participants, and participants received post-training follow-up sessions. The *Demand Sensitive* segment (22.9 %) was less intent on practice change. They preferred that individual teachers make practice change decisions, recommended discretionary coaching, and chose no post-training follow-up support. This study emphasizes the complex social, organizational, and policy context within which educators make practice change decisions. Efforts to disseminate strategies to improve the mental health outcomes of students need to be informed by the preferences of segments of educators who are sensitive to different dimensions of the practice change process. In the absence of a broad consensus of educators, administrators, and unions, potentially successful practice changes are unlikely to be adopted.

## Introduction

Schools represent an important context in which to prevent, identify, and intervene to reduce children’s mental health problems (Atkins, Hoagwood, Kutash, & Seidman, 2010; Stephan, Weist, Kataoka, Adelsheim, & Mills, 2007). Delivering mental health services via schools could reduce the barriers associated with clinics, balance targeted strategies with universal approaches, enhance transfer and maintenance, and improve service coordination and efficiency (Stephan et al., 2007). Although a growing number of evidence-based practices for improving the mental health outcomes of students are available (Hoagwood et al., 2007; Kratochwill et al., 2009), schools are sometimes slow to adopt these approaches (Atkins et al., 2010; Forman, Olin, Hoagwood, Crowe, & Saka, 2009). Efforts to introduce school-based mental health services fail when programs are incompatible with prevailing educational policies, practices, or philosophies, lack administrative backing, are inadequately funded, or provide insufficient training and follow-up support (Forman et al., 2009).

**Fig. 1:**
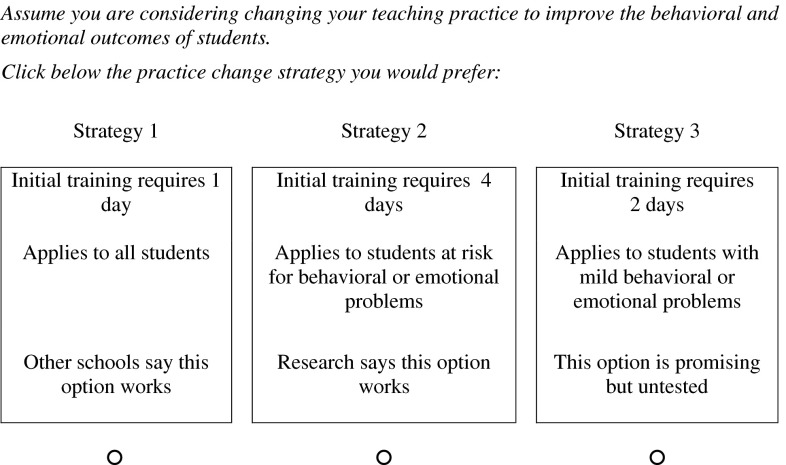
A sample of the format used in the 18 choice tasks completed by each participant. The study’s experimental design module randomly assigned one of 999 versions of the survey to each participant

Theory and research suggest that a strategy for implementing school-based mental health programs should be informed by the educators who might conduct these services (Gagnon, 2011; Jansson, Benoit, Casey, Phillips, & Burns, 2010). This study, therefore, used a discrete-choice conjoint experiment (DCE) to model the preferences of educators for the design of an approach to the implementation of evidence-based practices to improve the mental health of students. DCEs begin by defining practice change as a multi-attribute process. Attributes of the practice change process, for example, might include training time, coaching, or follow-up support. Each attribute has a range of levels. Training, for example, might require 1, 2, 3, or 4 days. An experimental design algorithm combines the study’s attribute levels into a set of hypothetical practice change options. Different combinations of these options are presented in a series of choice sets (Fig. 1). The choices respondents make allow investigators to estimate the relative importance of each attribute and the level of each attribute that is preferred.

Although this approach was proposed by mathematical psychologists (Luce & Tukey, 1964), DCEs have not been widely used by behavioral scientists. Marketing research, however, has adapted these methods to engage consumers in the service and product design process (Orme, 2009), while economists use DCEs to estimate the value of different outcomes and dimensions of the health service delivery process (Ryan, Gerard, & Amaya-Amaya, 2008). These methods have, only recently, been applied to the design of school-based prevention programs (Cunningham et al., 2009; Cunningham, Vaillancourt, Cunningham, Chen, & Ratcliffe, 2011), children’s mental health services (Cunningham et al., 2008; Cunningham et al., 2013; Waschbusch et al., 2011), and the dissemination of evidence-based mental health practices (Cunningham et al., 2012).

Developers must balance the trade-offs associated with design features that influence the acceptability, cost, and outcome of school-based mental health programs. Educators considering the adoption of evidence-based practices to improve mental health outcomes must weigh competing curriculum demands, time constraints, administrative policies, and educational philosophies (Teich, Robinson, & Weist, 2008). The multi-attribute trade-offs presented in DCEs approximate the complexity of the design decisions program developers make, reflect the real-world practice change choices educators consider, limit social desirability biases (Caruso, Rahnev, & Banaji, 2009; Goldberg Lillehoj, Griffin, & Spoth, 2004; Phillips, Johnson, & Maddala, 2002), and prompt the heuristics that are likely to influence professional development choices (Shah & Oppenheimer, 2008). DCEs can estimate the extent to which the components of a complex implementation strategy influence practice change decisions and simulate the response of educators to a range of design options prior to costly efforts to take programs to scale (Orme, 2009).

We began by examining three general research questions (RQ) regarding the mental health practice change preferences of educators. We then addressed two hypotheses regarding their response to several more specific approaches to the practice change process.

- RQ 1: Which practice change attributes influence the choices of educators?

Although a large number of factors are thought to influence the adoption of evidence-based practices (Damschroder et al., 2009; Forman et al., 2009; Rogers, 2003), we know little about the extent to which specific features of programs, the practice change process, or the social context in schools influences the decision to adopt mental health strategies. To explore this question, we estimated the relative influence of 16 four-level attributes on decisions regarding mental health practice change strategies. These included features of programs (e.g., universal versus targeted focus), attributes of the training process (e.g., variations in training time demands), and attributes of the social context in which educators make practice change decisions (e.g., variations in colleague support for different practice change options).

- RQ 2: Are there segments with different mental health practice change preferences?

Previous studies suggest segments of educators preferring different approaches to the design and implementation of school-based prevention programs (Cunningham et al., 2009). Aggregating data from educators with different views can contribute to misleading preference estimates and policy recommendations (Johnson & Mansfield, 2008). The current study, therefore, used latent class analysis (Lanza & Rhoades, 2013) to identify segments of educators having different practice change preferences.

- RQ 3: What are the demographic and attitudinal correlates of segment membership?

Next, we explored the demographics and attitudes associated with membership in segments preferring different approaches to practice change. Previous studies (Cunningham et al., 2009), for example, have linked practice change preferences to the attitudes reflected in the Theory of Planned Behavior, a model used widely to study health behavior change (Armitage & Conner, 2001). This model predicts that educators expecting: (1) greater benefits from practice change (attitudes), (2) more encouragement from colleagues and administrators (subjective norms), (3) fewer barriers to implementation, and (4) more success in their practice change efforts (perceived behavioral control), would be more likely to reside in segments intent on using the multi-faceted approaches needed to successfully implement evidence-based practices (Forman et al., 2009; Lochman et al., 2009). Although some evidence suggests professional development programs based on the Theory of Planned Behavior improve outcomes (Casper, 2007), few studies have applied this model to the study of mental health practice decisions (Perkins et al., 2007). We used a new measure to explore the relationship between the Theory of Planned Behavior’s subscales and membership in segments preferring different practice change strategies.

- Hypothesis 1: Educators will prefer small-group approaches to practice change

We used Randomized First Choice Simulations, a forecasting tool used widely in the field of marketing research (Orme, 2009), to estimate the percentage of educators likely to prefer three approaches to the mental health practice change process. As a standard, we modeled the large-group presentations that are widely used for school-based in-service education. Second, we modeled the response of educators to an enhanced small-group approach with the demonstration, practice, coaching, and follow-ups that are most likely to yield successful practice change (Forman et al., 2009; Lochman et al., 2009; Payne & Eckert, 2010). Third, the Internet affords a convenient, flexible, economical platform to support practice change decisions, disseminate evidence-based strategies, and organize communities of practice (Bernhardt, Mays, & Kreuter, 2011; Shafer, Rhode, & Chong, 2004; Sholomskas et al., 2005). Our simulations, therefore, included a practice change strategy delivered via the Internet. Given evidence that educators prefer active learning approaches to the acquisition of school-based prevention strategies (Cunningham et al., 2009), we predicted that most would prefer an enhanced small-group approach.

- Hypothesis 2: Educators will prefer local decision control

Governments are increasingly involved in the selection and dissemination of evidence-based mental health strategies (Schroeder et al., 2012). Decision control research, in contrast, suggests that top-down decisions would decrease support for practice change (Cunningham et al., 2002; De Cremer, Cornelis, & Van Hiel, 2008; Terwel, Harinck, Ellemers, & Daamen, 2010). To examine this question, we used Randomized First Choice simulations to predict the response of educators to practice change options that were selected by the provincial ministry of education versus chosen by local schools. Hypothesis 2 predicted that educators would prefer school-based practice change decisions.

## Methods

### Participants

The study protocol was approved by the university/hospital research ethics board. We stratified elementary schools in the publicly funded boards of education serving a Canadian community of 505,000 residents into socio-demographic quadrants. A random sample of 66 elementary and elementary middle schools was selected; 50 agreed to participate. Of the 1,228 educators working in these schools, 1,010 agreed to participate and provided complete data (82 %). Participants endorsed a consent assuring anonymity, the option not to participate, and the freedom to withdraw from the study. The demographics of the sample are described in Table 5. Most participants were women (80.9 %), employed as teachers (77.4 %), in non-secondary schools.

### Procedure

#### Attribute Development

To identify attributes of the mental health practice change process that were relevant, we conducted focus groups with a purposive sample of 24 administrators and 23 teachers (Bridges et al., 2011). Discussions were transcribed verbatim and coded to identify practice change themes. The results of this study are presented separately (Barwick et al., 2013). Themes were distilled to 16 practice change attributes each having four levels (Verlegh, Schifferstein, & Wittink, 2002). The study’s attributes are detailed in Tables 3, 4.

#### Survey Design

The Internet survey was programmed and fielded using SSI Web Version 7 (Sawtooth Software, 2013). Following a warm-up task, participants completed 17 choice sets. As depicted in Fig. 1, each set presented three practice change options. We used two strategies to limit the tendency to simplify based on a single must have or unacceptable level. First, according to a partial profile design, each of the three options in choice sets was described by the levels of three attributes (Patterson & Chrzan, 2003). Second, we employed a balanced overlap design allowing the same attribute level to appear in more than one choice-set option (Chrzan, Zepp, & White, 2010; Sawtooth Software, 2013). To minimize sequence or context effects, and to maximize efficiency, Sawtooth Software’s experimental design algorithm composed 999 versions (the software’s maximum) of the survey. This algorithm presents attribute levels independently (orthogonality) and ensures that each attribute’s levels appear approximately an equal number of times in each survey (balance). One version of the survey was randomly assigned to each respondent (Johnson et al., 2013).

#### Attitudes and Demographics

To identify attitudes that might be associated with membership in segments preferring different approaches to practice change, we developed a scale with 30 questions (1 = strongly disagree to 5 = strongly agree) measuring the components of the Theory of Planned Behavior (Armitage & Conner, 2001). This measure included subscales reflecting the benefits of improving mental health outcomes (attitudes), the extent to which supervisors, colleagues unions, parents, etc. would encourage the adoption of strategies to improve behavioral and emotional outcomes (subjective norms), the extent to which time, curriculum demands, resources etc. acted as barriers to practice change (perceived behavioral control barriers), the confidence that one has the knowledge and skill to improve behavioral and emotional outcomes (perceived behavioral control—self-efficacy), and an intent subscale reflecting a willingness to engage in practice change activities such as readings, workshops, and coaching. The internal consistency of the scale and sample items are presented in Table 1. Participants reported gender, age, educational level, role in the educational system, and years of experience.

**Table 1:** Description of Theory of Planned Behavior scale

Subscales	*Items*	*α* ^a^	Sample question content
Attitudes: anticipated benefits	7	0.97	Improving the emotional or behavioral outcome of students will: Improve the academic outcomes of students with behavioral or emotional problems
Subjective norms	8	0.84	Indicate whether you agree or disagree that each of the following would actually encourage your efforts to change your practice to improve the behavioral or emotional outcomes of students: My teaching colleagues
Perceived behavioral control: barriers	5	0.71	To what extent might each of the following make it difficult for you to change your practice to improve the behavioral or emotional outcome of students: There’s not enough time
Perceived behavioral control: self-efficacy	5	0.81	I am confident that I have the: Knowledge to improve behavioral or emotional outcomes
Intent	5	0.76	To improve the behavioral or emotional outcomes of students I would be willing to: Participate in a 1-day workshop to learn new skills

^a^ *α* = Cronbach’s alpha. The factor structure of the Theory of Planned Behavior scale and a complete listing of items are available upon request

### Data Analysis

We used version 4.5 of Latent Gold Choice (Vermunt & Magidson, 2005) to address RQs 1 and 2. This program uses conditional logit and latent class methods to: (1) identify segments of participants with different practice change preferences and (2) compute parameter estimates (zero-centered utility coefficients) quantifying each segment’s preference for the levels of each attribute. As a finite mixture model, this approach assumes that choices reflect the mental health practice change preferences of several unobserved (latent) segments of educators (Lanza & Rhoades, 2013; Vermunt & Magidson, 2005). Using a maximum likelihood criterion to predict the posterior probability of membership in each segment, we estimated solutions with 1, 2, 3, 4, and 5 latent classes or segments (Lanza & Rhoades, 2013; Vermunt & Magidson, 2005). Tolerance, the point at which iterations terminate, was set at 1.0 × 10^−8^. Latent class analyses may yield unrepresentative solutions (local maxima). Each solution, therefore, was replicated 10 times beginning at randomly selected starting points (Lanza & Rhoades, 2013). Because covariates can improve latent class models (Huang & Bandeen-Roche, 2004; Yang & Yang, 2007), we included two variables (sex and the intent to engage in practice change activities), in the latent class solution. Importance scores were derived by dividing the range of each attribute’s utility coefficients by the summed utility coefficient range of all attributes (Orme, 2009). Importance scores show the relative influence of variations in the levels of each attribute on practice change choices; attributes with higher scores exert a greater influence.

We used Chi square to examine the demographics associated with membership in each latent class segment and used a one-way MANOVA with univariate ANOVAs to compare the Theory of Planned Behavior subscale scores of educators in different segments. Partial ETA squared was computed as a measure of the strength of the relationship between segment membership and Theory of Planned Behavior subscale scores.

We used Sawtooth Software’s Randomized First Choice Simulator (Huber, Orme, & Miller, 2007; Orme & Huber, 2000) to examine Hypotheses 1 and 2. Using informant-level utility coefficients (Vermunt & Magidson, 2005), we predicted each participant’s response to the combinations of attribute levels that approximate the complex practice change strategies educators planning to improve mental health outcomes might actually consider. 200,000 sampling iterations were computed to model error in our estimations. Simulations assume that educators would choose an approach to practice change that maximizes utility. 1

## Results

- RQ 1: Which practice change attributes influence the choices of educators?
- RQ 2: Are there segments with different mental health practice change preferences?

As shown in Table 2, fit criteria often yield conflicting recommendations regarding the number of segments that should be included in latent class solutions (Nylund, Asparouhov, & Muthén, 2007). The Akaike Information Criterion, for example, tends to overestimate the number of segments known to exist in simulated data sets (Nylund et al., 2007). The Bayesian Information Criterion (BIC), which imposes a more stringent correction for the number of parameters estimated, provides more accurate estimates (Nylund et al., 2007). A two-segment solution (Table 2) yielded the lowest BIC and Consistent Akaike Information Criteria (CAIC) values (Nylund et al., 2007). A bootstrap −2 log-likelihood difference test (Vermunt & Magidson, 2005) confirmed that a two-segment solution was a statistically significant improvement over a one-class model (470.13, *p* < 0.001). The two-segment solution provided a pattern of utility coefficients that was easily interpreted and segments large enough to inform real-world practice change (Lanza & Rhoades, 2013; Orme, 2009).

**Table 2:** Fit indices for 1–5 class latent solutions

Measure	Number of latent classes				
	1	2	3	4	5
Parameters estimated	48	99	150	201	252
Degrees of freedom	962	911	860	809	758
Log-likelihood (LL)	−12,557.97	−12,322.90	−12,201.87	−12,116.45	−12,043.33
Log-prior	−1.48	−2.46	−2.98	−3.35	−3.58
Log-posterior	−12,559.44	−12,325.36	−12,204.85	−12,119.79	−12,046.90
AIC (based on LL)	25,211.93	24,843.81	24,703.74	24,634.89	24,590.66
AIC3 (based on LL)	25,259.93	24,942.81	24,853.74	24,835.89	24,842.66
BIC (based on LL)	25,447.98	25,330.66	25,441.39	25,623.35	25,829.92
CAIC (based on LL)	25,495.98	25,429.66	25,591.39	25,824.35	26,081.92
Entropy *R* ^2^	1	0.58	0.55	0.61	0.62

*BIC* Bayesian Information Criterion, *AIC* Akaike Information Criterion, *CAIC* Consistent Akaike Information Criterion

Entropy *R* ^2^ values range from 0 to 1 with higher values reflecting a greater separation of segments

Importance scores (Table 3), utility coefficients, and associated *z* values (Table 4) show that the two segments agreed on the relative value of many of the study’s 16 practice change attributes, an observation reflected in entropy scores (Table 2). Their views regarding a set of strategically important design features, however, were quite different. We begin by examining the relative importance of those attributes on which the two segments agreed. Attributes that distinguished the segments are then considered.

**Table 3:** Relative importance of practice change attributes to the Change Ready, and Demand Sensitive segments

Attribute	Latent class segment						*F*	*η* ^2^
	Change Ready			Demand Sensitive				
	*R*	*M*	(SD)	*R*	*M*	(SD)		
*Contextual and social attributes*								
Presenter’s qualities	1	**11.6**	(0.2)	2	10.8	(0.6)	866.5***	0.5
Colleague support	2	10.0	(0.1)	3	**10.3**	(0.2)	521.7***	0.3
Union endorsement	5	9.0	(0.2)	4	**10.0**	(0.1)	7,412.4***	0.9
Compatibility with practice	3	9.7	(0.2)	1	**11.4**	(0.3)	8,872.8***	0.9
Administrative support	4	**9.2**	(0.0)	5	8.8	(0.4)	805.1***	0.4
Provincial curriculum links	8	6.5	(0.3)	6	**8.6**	(0.5)	8,382.6***	0.9
*Content attributes*								
Supporting evidence	7	**6.6**	(0.1)	9	5.3	(0.6)	3,708.8***	0.8
Focus on knowledge versus skills	10	**4.9**	(0.1)	13	3.1	(0.7)	5,264.1***	0.8
Observability, trialability	12	**4.8**	(0.3)	16	2.5	(0.5)	9,054.9***	0.9
Universal versus targeted	15	**2.5**	(0.0)	15	2.4	(0.1)	510.6***	0.3
*Practice change process attributes*								
Coaching to improve skills	6	**7.5**	(0.3)	10	4.5	(0.7)	7,625.3***	0.9
Workshop size	9	**6.0**	(0.0)	8	5.4	(0.4)	2,288.6***	0.7
Follow-up support	11	**4.9**	(0.5)	12	3.8	(1.4)	344.6***	0.3
Training time demands	13	3.0	(0.4)	7	**6.6**	(1.0)	7,123.4***	0.9
Selection process	14	2.9	(0.1)	11	**3.8**	(0.4)	3,730.0***	0.8
Internet options	16	1.0	(0.2)	14	**2.8**	(0.6)	5,789.5***	0.9

Importance scores for each participant were derived by converting the range of each attribute’s levels to a percentage of the sum of the utility value ranges of all 16 attributes. Higher importance scores show that experimental variations in the levels of that attribute exerted a greater influence on practice change choices. For each attribute, the segment with the highest importance score is *bolded*

*R* = relative rank of importance score; *M* = mean importance score value; (SD) = standard deviation

*** *p* < 0.001

**Table 4:** Standardized (zero-centered) utility coefficients and *Z* values reflecting preference of the Change Ready and Demand Sensitive segments for the levels of each attribute

Attribute	Latent class segment				*Wald*
	Change Ready		Demand Sensitive		
	*U*	*Z*	*U*	*Z*	
*Contextual and social attributes*					
Presenter’s qualities					61.49***
Trainer is not engaging nor an expert	−1.73	−18.68	−0.77	−6.49	
Trainer is engaging but not an expert	−0.02	−0.32	0.19	1.88	
Trainer is an expert but not engaging	−0.13	−1.82	−0.29	−2.54	
Trainer is an engaging expert	**1.88**	**22.93**	**0.87**	**7.12**	
Colleague support					34.25***
0 % of my colleagues support this option	−1.80	−18.71	−0.93	−7.38	
33 % of my colleagues support this option	−0.21	−3.22	−0.15	−1.48	
67 % of my colleagues support this option	0.70	11.03	0.41	3.94	
100 % of my colleagues support this option	**1.31**	**19.70**	**0.67**	**5.98**	
Compatibility with practice					19.47***
Is 0 % compatible with my practice	−1.76	−18.64	−1.04	−7.96	
Is 33 % compatible with my practice	−0.17	−2.67	−0.11	−1.04	
Is 67 % compatible with my practice	0.72	11.44	0.35	3.38	
Is 100 % compatible with my practice	**1.22**	**18.13**	**0.81**	**7.22**	
Union endorsement					23.39***
The union does not support this option	−1.64	−18.64	−0.91	−7.35	
The union supports this option 33 %	−0.09	−1.39	−0.08	−0.74	
The union supports this option 67 %	0.58	9.63	0.31	3.09	
The union supports this option 100 %	**1.15**	**17.08**	**0.70**	**6.63**	
Administrative support					35.76***
My school’s administrator(s) don’t support this option	−1.62	−18.17	−0.82	−6.62	
My school’s administrator(s) support this option 33 %	−0.18	−2.89	−0.09	−0.89	
My school’s administrator(s) support this option 67 %	0.54	8.83	0.40	4.09	
My school’s administrator(s) support this option 100 %	**1.26**	**19.48**	**0.51**	**4.62**	
Provincial curriculum links					8.62*
Content linked 0 % to the provincial curriculum	−1.17	−15.68	−0.93	−6.95	
Content linked 33 % to the provincial curriculum	−0.20	−3.11	0.07	0.69	
Content linked 67 % to the provincial curriculum	0.54	9.55	0.32	3.15	
Content linked 100 % to the provincial curriculum	**0.82**	**12.87**	**0.53**	**4.83**	
*Content attributes*					
Supporting evidence					34.84***
This option is promising but untested	−1.20	−15.40	−0.46	−3.94	
Other schools say this option works	0.07	1.21	0.24	2.54	
Research says this option works	0.24	4.14	−0.07	−0.69	
Research and other schools say this option works	**0.89**	**14.20**	**0.29**	**2.67**	
Focus on knowledge versus skill					29.37***
100 % focus on knowledge	−1.03	−13.70	−0.21	−1.82	
67 % focus on knowledge, 33 % on step-by-step skills	0.08	1.37	−0.10	−0.98	
33 % focus on knowledge, 67 % on step-by-step skills	**0.54**	**8.70**	0.15	1.36	
100 % focus on step-by-step skills	0.41	6.77	**0.17**	**1.54**	
Observability and trialability					41.74***
I have not tried or seen this work	−0.92	−11.81	−0.09	−0.71	
I have seen this work	−0.02	−0.34	**0.21**	**2.20**	
I tried this and it worked for me	0.31	5.10	−0.15	−1.42	
I have seen this work, tried it, and it worked for me	**0.63**	**10.06**	0.02	0.22	
Universal versus targeted (applies to)					6.79
All students	**0.51**	**8.40**	**0.17**	**1.63**	
Students at risk for behavioral or emotional problems	0.01	0.13	0.11	1.11	
Students with mild behavioral or emotional problems	−0.28	−4.63	−0.08	−0.75	
Students with severe behavioral or emotional problems	−0.23	−3.80	−0.21	−1.97	
*Practice change process attributes*					
Coaching to improve skills					78.32***
No coaching	−1.57	−17.40	−0.38	−3.25	
If I want, I would get coaching to improve my skills	0.41	6.89	0.21	2.10	
If I need it, I would get coaching to improve my skills	0.33	5.47	**0.23**	**2.37**	
All participants get coaching to improve skills	**0.83**	**12.90**	−0.06	−0.55	
Workshop size					26.63***
I learn this alone	−0.89	−12.29	−0.38	−3.29	
I learn this in a group of 10	**0.98**	**15.92**	**0.41**	**3.79**	
I learn this in a group of 50	0.30	5.02	0.06	0.61	
I learn this in a group of 100	−0.39	−6.30	−0.09	−0.92	
Follow-up support					148.03***
Includes no training follow-up sessions	−1.15	−12.97	**0.42**	**3.68**	
Includes one 1-h training follow-up session	0.30	5.04	0.15	1.53	
Includes two 1-h training follow-up sessions	**0.49**	**8.24**	−0.11	−1.03	
Includes three 1-h training follow-up sessions	0.36	5.87	−0.46	−4.17	
Training time demands					5.47
Initial training requires 1 day	**0.36**	**6.19**	**0.66**	**6.02**	
Initial training requires 2 days	0.29	4.99	0.25	2.41	
Initial training requires 3 days	−0.14	−2.39	−0.35	−3.20	
Initial training requires 4 days	−0.51	−8.51	−0.56	−4.92	
Selection process					9.26*
Provincial ministry decides on this option	−0.48	−7.66	−0.30	−2.62	
Boards of education decide on this option	−0.02	−0.40	−0.30	−2.57	
Individual schools decide on this option	**0.40**	**7.06**	0.22	2.16	
Individual teachers decide on this option	0.10	1.64	**0.38**	**3.40**	
Internet options (training includes:)					10.44*
No Internet options	−0.11	−1.74	**0.27**	**2.55**	
Internet learning activities	**0.14**	**2.51**	0.22	2.18	
A moderated Internet discussion group	−0.13	−2.33	−0.28	−2.83	
Internet learning + moderated Internet discussion group	0.10	1.65	−0.20	−1.91	

U = parameter estimates expressed as zero-centered utility coefficients. Higher utility coefficients reflect a stronger preference. Z = Z scores (U/SE). SE = U/Z. Within segments, the highest attribute with the highest utility coefficient and *Z* value is *bolded*. *Z* values of 1.96 differ from zero (*p* < 0.05)

* *p* < 0.05; ** *p* < 0.01; *** *p* < 0.001

### Mental Health Practice Change Attributes Both Segments Preferred

Importance scores (Table 3) show that variations in the qualities of workshop presenters exerted an especially strong influence on practice change decisions. Utility coefficients (Table 4) suggest both segments preferred workshops conducted by engaging expert presenters. Contextual and social attributes, such as the support of colleagues, administrators, and unions, also exerted a relatively strong influence on practice change decisions (Table 3). Utility coefficients (Table 4) show that both segments preferred practice changes with 100 % support, 100 % compatibility, and close links to the provincial curriculum. Both segments preferred one-day, small-group workshops focusing on strategies for improving the mental health of all students, rather than those specifically applicable to children with behavioral or emotional problems. Importance scores (Table 3) suggest that variations in the availability of Internet options exerted very little influence on choices.

### Change Ready Educators

This segment, 77.1 % of the sample, preferred a selection process in which schools, rather than individual teachers or ministries, made decisions regarding mental health practice changes (Table 4). They were more likely to select an approach supported by both research and the views of other schools. Given conflicting evidence, they preferred options supported by research rather than the experience of other schools. Change Ready educators were more likely to adopt strategies they had observed, tried, and found to work. They preferred an emphasis on skills rather than knowledge. This segment thought all educators should receive coaching and preferred two one-hour training follow-up sessions. Although they were willing to pursue Internet learning activities, they were not interested in a moderated Internet discussion group.

### Demand Sensitive Educators

This segment, 22.9 % of the sample, preferred a program selection process in which individual teachers, rather than schools, boards of education, or ministries, made practice change decisions. Research exerted little influence on their practice change decisions. Faced with conflicting evidence, the views of other schools exerted a stronger influence on their choices than research. They preferred to simply observe, rather than try, new strategies. Instead of mandatory implementation coaching, this segment preferred that coaching was provided as needed. Demand Sensitive educators preferred that, once training was complete, no follow-up sessions were required. They preferred no Internet learning or moderated Internet discussion groups.

- RQ 3: What are the demographic and attitudinal correlates of segment membership?

The demographics of the two segments are summarized in Table 5. Covariate analysis showed that membership in the Change Ready segment was associated with higher intent scores, *z* = 6.7, Wald = 44.87, *p* < 0.001. Women were more likely to be members of the Change Ready segment; men were more likely to be in the Demand Sensitive segment, *z* = 4.03, Wald = 16.20, *p* < 0.001. A secondary MANOVA across Theory of Planned Behavior scores showed significant segment effects, *F* (−5, 1,004) = 39.67, *p* < 0.001. Table 6 shows that, in comparison to the Demand Sensitive segment, Change Ready educators anticipated more benefits to practice change, expected more normative encouragement, were more confident in their ability to accomplish change, and more intent on practice change. Perceived barriers were not associated with segment membership.

**Table 5:** Demographics percentages for the Change Ready and Demand Sensitive segments

	*N*	%	Latent class segment		*χ* ^2^
			Change Ready	Demand Sensitive	
Sample size	1,010	100	779	231	
Gender					52.0***
Male	193	19.1	57.5	42.5	
Female	817	80.9	81.8	18.2	
Age					15.5***
18–29	118	11.7	77.1	22.9	
30–39	342	33.9	70.8	29.2	
40–49	301	29.8	78.4	21.6	
50 or older	249	24.7	84.3	15.7	
Education					4.1
High school or less	6	0.6	50.0	50.0	
Some college or university	25	2.5	72.0	28.0	
College diploma or degree	110	10.9	79.1	20.9	
BA or BSc Degree	700	69.4	78.0	22.0	
Masters and PhD degree	168	16.6	74.4	25.6	
Work background					6.6
Principal and vice-principal	37	3.7	89.2	10.8	
Teacher	782	77.4	76.2	23.8	
Educational assistant	127	12.6	77.2	22.8	
Allied professionals	51	5.1	86.3	13.7	
Non-teaching support staff	12	1.2	66.7	33.3	
Divisions assigned (check all that apply)					
Preschool/JK	243	24.1	86.0	14.0	14.1***
Primary/junior	808	80.0	78.7	21.3	5.2*
Intermediate	367	36.3	76.8	23.2	0.0
Secondary	16	1.6	87.5	12.5	1.0
Years experience					18.1***
0–5 years	204	20.2	78.9	21.1	
6–10 years	239	23.7	74.5	25.5	
11–15 years	214	21.2	72.4	27.6	
16–20 years	114	11.3	70.2	29.8	
21 or more years	238	23.6	86.1	13.9	

* *p* < 0.05;** *p* < 0.01;*** *p* < 0.001

**Table 6:** Theory of Planned Behavior scale scores

Content of question	Latent class segment				*F*	*η* ^2^
	Change Ready		Demand Sensitive			
	*M*	(*SD*)	*M*	(*SD*)		
Attitudes (benefits)	30.3	(5.6)	29.1	(6.0)	7.03**	0.007
Subjective norms	30.6	(4.2)	28.9	(5.0)	26.56***	0.026
Perceived behavioral control: self-efficacy	19.5	(3.0)	18.8	(3.3)	7.42**	0.007
Perceived behavioral control: barriers	17.4	(3.5)	17.6	(3.6)	0.33	0.000
Intent	19.1	(3.2)	15.7	(3.5)	189.70***	0.158

*M* = mean; *SD* = standard deviation; *η* ^2^ = ETA^2^

* *p* < 0.05; ** p < 0.01; *** *p* < 0.001

- Hypothesis 1: Educators will prefer enhanced small-group practice change

Simulation 1 (Table 7) predicted the response of participants to three practice change strategies. We manipulated the levels of six attributes while holding the levels of ten attributes constant. *Standard Dissemination* was a (1) one-day, (2) large group (*n* = 50), with (3) no Internet options (4) focusing more on knowledge (67 %) than skills (33 %), with (5) no coaching, nor (6) follow-up support. The *Enhanced* option was a (1) three-day, (2) small group (*n* = 10), with (3) no Internet options (4) focusing more on skills (67 %) than knowledge (33 %), with (5) coaching for all participants, and (6) three one-hour follow-up sessions. The *Internet* option required (1) 3 days, (2) was pursued individually, via (3) Internet learning activities and a moderated Internet discussion group (4) focused 67 % on skills and 33 % on knowledge, with (5) no coaching, and (6) three one-hour Internet follow-up sessions. Simulation 1 predicted most Change Ready educators (98.4 %) would prefer an *Enhanced* approach to practice change (Table 7). Demand Sensitive participants, in contrast, would prefer the *Standard* option (53.3 %). Overall, few educators (0.6 %) were predicted to prefer an *Internet* approach.

**Table 7:** Randomized First Choice simulations: percentage of participants in each segment predicted to prefer different approaches to the dissemination of mental health practice change

Simulation	Total sample		Latent class segment			
			Change Ready		Demand Sensitive	
Practice change option	%	*(SE)*	%	*(SE)*	%	*(SE)*
*Simulation 1*						
Standard dissemination	12.8	(0.7)	1.4	(0.1)	53.3	(1.3)
Enhanced dissemination	86.5	(0.8)	98.4	(0.1)	44.8	(1.3)
Internet dissemination	0.6	(0.0)	0.3	(0.0)	1.9	(0.1)
*Simulation 2*						
Standard dissemination	17.0	(0.8)	3.6	(0.2)	64.4	(1.2)
Mandated enhanced dissemination	81.2	(0.9)	95.0	(0.2)	32.7	(1.1)
Internet dissemination	1.7	(0.0)	1.4	(0.0)	2.9	(0.1)

- Hypothesis 2: Educators will prefer local decision control

Next, we tested the prediction that educators would prefer practice changes selected by individual schools rather than government ministries. We manipulated the levels of seven attributes while holding nine constant. *Standard Dissemination* was a (1) one-day, (2) large group (*n* = 50), with (3) no Internet options (4) focusing more on knowledge (67 %) than skills (33 %), with (5) no coaching, nor (6) follow-up support that was (7) selected by individual schools. The *Enhanced* option was a (1) three-day, (2) small group (*n* = 10), with (3) no Internet options (4) focusing more on skills (67 %) than knowledge (33 %), with (5) coaching for all participants, and (6) three one-hour follow-ups that was (7) selected by the provincial ministry of education. The *Internet* option required (1) 3 days, (2) was pursued individually, via (3) Internet learning activities and a moderated Internet discussion group (4) focused 67 % on skills and 33 % on knowledge, with (5) no coaching, and (6) three one-hour Internet follow-up sessions, (7) selected by individual schools. Simulation 2 (Table 7) predicted that a provincial selection process would exert a limited influence on practice change preferences, reducing the Change Ready educators preference for the *Enhanced* option from 98.4 % (Simulation 1) to 95.0 % (Simulation 2) and the Demand Sensitive segment’s preference for the *Enhanced* option from 44.8 to 32.7 %.

## Discussion

We modeled the conditions under which educators would adopt practice changes to improve the mental health outcomes of students. Latent class analysis is a probabilistic method that recognizes the overlapping nature of preferences (Lanza & Rhoades, 2013). Both segments chose small-group workshops conducted by engaging expert trainers. They preferred a focus on step-by-step skills that were applicable to all students, consistent with the provincial curriculum, compatible with current practice, and proven effective via both research and the experience of other schools. Educators were especially sensitive to the support of colleagues, administrators, and unions.

The pattern of overlapping preferences observed here has been reported in studies of the prevention program preferences of educators (Cunningham et al., 2009) and students (Cunningham et al., 2011), the knowledge translation preferences of mental health professionals (Cunningham et al., 2012), and the service preferences of parents of children with mental health problems (Cunningham et al., 2008, 2013; Waschbusch et al., 2011). Despite general agreement on the relative importance of many attributes of the practice change process, however, views regarding a set of strategically important design features differed. Below we summarize differences in the preferences of the two segments and consider the implications of our findings.

The Change Ready segment, 77.1 % of the sample, anticipated more benefits to practice change, expected more encouragement to participate, and expressed more confidence in their ability to accomplish change. As the Theory of Planned Behavior would predict (Armitage & Conner, 2001), they reported a greater intent to change. This segment preferred that schools select practice changes. They valued the coaching and follow-up support that has been linked to successful implementation (Payne & Eckert, 2010). This segment’s preferences for school-based decision making is similar to those of the Decision Sensitive educators described previously (Cunningham et al., 2009).

In comparison to Change Ready educators, the Demand Sensitive segment (22.9 %) expected fewer benefits to mental health practice change, anticipated less support, were less confident in their ability to accomplish change, and were less intent on pursuing practice change. They preferred that individual educators make practice change decisions, rejected mandatory coaching, were not interested in Internet learning options, and chose not to receive follow-up support. Simulations predicted they would be least likely to choose an *Enhanced* strategy with the coaching and follow-up sessions that would increase the likelihood of successful practice change. This segment’s size and sensitivity to practice change time demands is consistent with the prevention program design preferences of Cost Sensitive educators in a previous study (Cunningham et al., 2009).

### Implications

#### Conduct Practice Change in Small Groups

A range of technology-enabled approaches to the dissemination and implementation of evidence-based practices has been proposed (Bernhardt et al., 2011; Shafer et al., 2004; Sholomskas et al., 2005). One might expect that the convenient, flexible learning options afforded by the Internet would appeal to educators, particularly those in the Demand Sensitive segment (Bernhardt et al., 2011). Simulations, nonetheless, suggested that, in comparison to small-group training, a relatively small percentage of this study’s participants would adopt a practice change strategy using the Internet as either a learning option or mechanism for supporting implementation. Two factors may contribute to this finding. First, this study emphasizes the importance of the social context within which educators make practice change decisions. The preference for small-group learning conducted by engaging experts suggests professional development serves an important social function for educators. The Internet may not approximate the quality of the face-to-face interaction that can be achieved by engaging workshop leaders. Second, educators preferred training focusing on the acquisition of step-by-step skills. Although the Internet may be useful for conveying new knowledge, it may be less effective in promoting the acquisition of the skills that were of interest to educators (Shafer et al., 2004).

#### Enable School-Based Decisions

Governments are increasingly involved in the selection of evidence-based mental health strategies (Schroeder et al., 2012). Educators, in contrast, preferred individual or school-based decisions rather than government-selected initiatives. This finding is consistent with evidence that local decisions improve the implementation of school-based prevention programs (Payne, Gottfredson, & Gottfredson, 2006) and with a wider body of organizational decision control research (Cunningham et al., 2002; De Cremer et al., 2008; Terwel et al., 2010). Simulations, however, suggest that, in comparison to the qualities of presenters, compatibility with current practices, and the support of colleagues, administrators, and unions, the process via which practice changes were selected exerted a relatively limited influence on practice change preferences. Boards of education, nonetheless, could encourage local decision control by enabling schools to select from a menu of evidence-based options supported by the combination of scientific evidence and real-world effectiveness educators valued.

#### Build a Practice Change Consensus

Diffusion theory emphasizes the role that early starters play in practice innovation (Rogers, 2003). Although Change Ready educators might adopt evidence-based practices earlier than educators in the Demand Sensitive segment, their sensitivity to the views of colleagues, administrators, and unions suggests that sustaining these changes will require a broad consensus. In contrast to a government selection process, the school-based decisions preferred by many educators could build the consensus needed to support practice change (Forman et al., 2009). The importance of broader contextual support is consistent with qualitative findings (Massey, Armstrong, Boroughs, Henson, & McCash, 2005), and quantitative studies linking this factor to the successful implementation of school-based programs (Gregory, Henry, & Schoeny, 2007; Payne et al., 2006).

#### Engage the Demand Sensitive Segment

Demand Sensitive educators were less intent on pursuing mental health practice change and willing to invest less time in the follow-up activities associated with successful implementation (Forman et al., 2009; Gottfredson & Gottfredson, 2002; Han & Weiss, 2005; Hanley et al., 2009; Lochman et al., 2009). Simulations predicted that more than 53.3 % of this segment would choose a standard presentation that lacked extended training, hands-on learning opportunities, coaching, and longer-term support. Although Demand Sensitive educators would constitute a small proportion of the staff in an individual school, our findings suggest that, given the sensitivity of educators to the views of their colleagues, the failure to engage this segment could compromise the implementation of school-wide mental health strategies.

How can schools engage the Demand Sensitive segment? First, this segment was more likely to support a selection process that maximized either personal or local decision control. It is important, therefore, to ensure that opinion leaders from this segment are included in practice change decisions (Atkins et al., 2008). Second, given this segment’s sensitivity to follow-up time demands, the components (Weisz et al., 2011) of a complex mental health strategy could be introduced sequentially via brief skill-focused workshops integrated into the professional development days that are part of educational plans of many schools. Third, conducting training in the small groups preferred by most participants would allow planners to align practice change processes with the preferences of different segments. Fourth, Demand Sensitive educators anticipated fewer benefits, less social support, and reported lower change self-efficacy than Change Ready educators. The Theory of Planned Behavior predicts that providing evidence supporting the benefits of practice change, mobilizing the support of colleagues, and adopting an approach enhancing practice change self-efficacy would increase this segment’s intent to participate (Armitage & Conner, 2001).

## Limitations

This study was conducted in Canada. Our results require replication in other settings.

Second, we sampled elementary and elementary/intermediate schools. The practice change preferences of secondary school educators require further study. Third, we studied attributes influencing the adoption of a practice change strategy. Attributes influencing educator’s longer-term commitment to a new approach may differ. Finally, our simulations are based on utility coefficients derived from hypothetical practice change choices. Although stated intentions are a moderately good predictor of actual behavior (Armitage & Conner, 2001), real-world tests of our simulations are required.

## Conclusion

This study emphasizes the complex social, organizational, and policy context influencing the adoption of school-based mental health practice changes. Efforts to introduce strategies to improve the mental health outcomes of students need to consider the preferences of segments of educators who are sensitive to different dimensions of the practice change process. In the absence of an approach supported by a broad consensus of educators, administrators, and unions, potentially successful practice changes are unlikely to be adopted or sustained.

## Footnotes

1 To estimate the predictive validity of simulations, “hold-out” choice sets were presented at positions 5 and 12 in the choice set sequence (Orme, 2009). Although the format of hold-out choice sets was similar to the example depicted in Fig. 1, the combination of attribute levels included in hold-out choice sets was identical for all participants. Hold-out choices were not used to estimate the utility coefficients in Table 4. We computed Randomized First Choice simulations using the remaining 15 choice sets to predict responses to the two hold-out choice sets. The small discrepancy between simulated and observed responses (mean absolute errors = 3.2 and 2.1) suggests predictive validity was satisfactory.
